# Human Kinase IGF1R/IR Inhibitor Linsitinib Controls the In Vitro and Intracellular Growth of *Mycobacterium tuberculosis*

**DOI:** 10.1021/acsinfecdis.2c00278

**Published:** 2022-09-01

**Authors:** Heng Wang, Jing Bi, Yuan Zhang, Miaomiao Pan, Qinglong Guo, Genhui Xiao, Yumeng Cui, Song Hu, Chi Kin Chan, Ying Yuan, Takushi Kaneko, Guoliang Zhang, Shawn Chen

**Affiliations:** Global Health Drug Discovery Institute, Haidian, Beijing 100192, China; National Clinical Research Center for Infectious Diseases, Guangdong Provincial Clinical Research Center for Tuberculosis, Shenzhen Third People’s Hospital, Southern University of Science and Technology, Shenzhen 518112, China; Global Health Drug Discovery Institute, Haidian, Beijing 100192, China; Global Health Drug Discovery Institute, Haidian, Beijing 100192, China; National Clinical Research Center for Infectious Diseases, Guangdong Provincial Clinical Research Center for Tuberculosis, Shenzhen Third People’s Hospital, Southern University of Science and Technology, Shenzhen 518112, China; Global Health Drug Discovery Institute, Haidian, Beijing 100192, China; Global Health Drug Discovery Institute, Haidian, Beijing 100192, China; Global Health Drug Discovery Institute, Haidian, Beijing 100192, China; Global Health Drug Discovery Institute, Haidian, Beijing 100192, China; Global Health Drug Discovery Institute, Haidian, Beijing 100192, China; Global Alliance for TB Drug Development, New York, New York 10005, United States; National Clinical Research Center for Infectious Diseases, Guangdong Provincial Clinical Research Center for Tuberculosis, Shenzhen Third People’s Hospital, Southern University of Science and Technology, Shenzhen 518112, China; Global Health Drug Discovery Institute, Haidian, Beijing 100192, China

**Keywords:** *M. tuberculosis*, *glutamine synthetase*, IGF1R/IR inhibitor, autophagy, ATP-dependent enzyme

## Abstract

ATP provides energy in the biosynthesis of cellular metabolites as well as regulates protein functions through phosphorylation. Many ATP-dependent enzymes are antibacterial and anticancer targets including human kinases acted on by most of the successful drugs. In search of new chemotherapeutics for tuberculosis (TB), we screened repurposing compounds against the essential glutamine synthase (GlnA1) of *Mycobacterium tuberculosis* (Mtb) and identified linsitinib, a clinical-stage drug originally targeting kinase IGF1R/IR as a potent GlnA1 inhibitor. Linsitinib has direct antimycobacterial activity. Biochemical, molecular modeling, and target engagement analyses revealed the inhibition is ATP-competitive and specific in Mtb. Linsitinib also improves autophagy flux in both Mtb-infected and uninfected THP1 macrophages, as demonstrated by the decreased p-mTOR and p62 and the increased lipid-bound LC3B-II and autophagosome forming puncta. Linsitinib-mediated autophagy reduces intracellular growth of wild-type and isoniazid-resistant Mtb alone or in combination with bedaquiline. We have demonstrated that an IGF-IR/IR inhibitor can potentially be used to treat TB. Our study reinforces the concept of targeting ATP-dependent enzymes for novel anti-TB therapy.

## Graphical Abstract

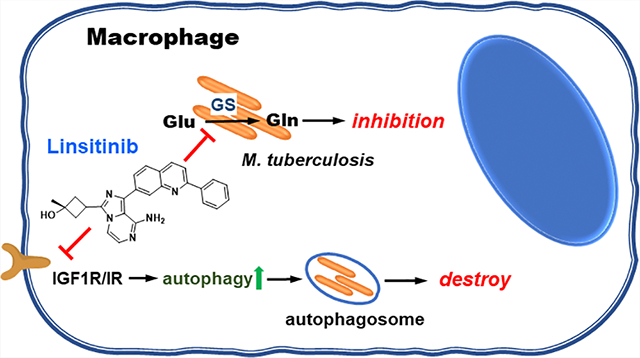

Tuberculosis (TB) caused by *Mycobacterium tuberculosis* (Mtb), one of the world’s top infectious killers, is a major public health threat. About 9.9 million people contracted TB in 2020, and 1.5 million ultimately died.^1^ The World Health Organization has been promoting research and innovation to end the TB epidemic by 2030. However, the coronavirus pandemic has significantly slowed the progress made in the battle against TB. The increased resistance and emergence of multidrug-resistant Mtb strains have often rendered the existing TB treatment regimens ineffective. New antibiotics and novel anti-TB therapies are urgently needed.

Traditional anti-TB drug discovery starts with phenotypic or target-based approaches. The phenotypic approach involves observational empirical evidence while the target-based approach is rational and hypothesis-driven. The phenotypic approach has proven to be more successful in antibacterial discovery but suffers from problems in hit validation. Host-directed therapy (HDT) is an emerging modality of treating infectious diseases.^2–4^ HDT for TB utilizes small molecules to modulate the host response for the benefit of improving the efficacy of anti-TB drugs against drug-resistant nonreplicating bacteria, shortening current antibiotic treatments, etc.^5,6^ Although HDT as adjuvant therapy often makes full use of approved drugs, new technologies, such as high-content imaging-based screening of large libraries made up of the repurposing drugs and even more drug-like compounds against intracellular Mtb, have opened an avenue for discovery of novel anti-TB/HDT leads.^7,8^ Furthermore, common TB drugs, including isoniazid (INH), rifampicin (RIF), and bedaquiline (BDQ) with defined bacterial targets, have all been shown to have host-targeting mechanisms.^9–11^ Thus, it is reasonable to identify anti-TB and HDT dual-targeting hits especially when screening with pharmacologically active compounds.

Human kinase inhibitors can be applied in the anti-infective area as HDT and/or a directly acting antibacterial agent.^12,13^ Kinase catalyzes the transfer of a phosphate group from ATP to specific proteins or small biomolecules. After a macrophage engulfs Mtb to form subcellular phagosome, kinase activities regulate the autophagy defense system and transduce metabolic signals favorable or unfavorable to intracellular Mtb. Interestingly, the Mtb genome encodes 11 eukaryotic-like serine/threonine protein kinases,^14^ among which PknA and PknB are essential for Mtb growth and PknG is indispensable for virulence. Early efforts searched for kinase inhibitors targeting the bacterial and host cell signaling pathways.^15,16^ Ongoing research focuses on these bacterial kinases as anti-TB targets.^17^ Phenotypic screening of ~26,000 select kinase-type inhibitors identified hundreds of hits with significant in vitro anti-TB activity.^18^ Another pioneering effort is the modification of a human tyrosine kinase inhibitor for a new ATP-competitive inhibitor of bacterial D-alanine-D-alanine ligase, which is a target validated by the anti-TB drug D-cycloserine.^19^ In addition to the phosphorylating enzymes, ATP-dependent enzymes also include adenylating enzymes that activate carboxylic acids to an acyladenylate intermediate. The acyl group is subsequently transferred to a nucleophilic group to make amide, ester, or thioester metabolites that could be critical for Mtb growth or survival.^20^ A proteomic profiling of Mtb led to the finding of 122 proteins with ATP-binding properties, roughly 60% of which are reported to be essential.^21^ On the other hand, imaging or cell-sorting-based screenings with repurposing compound libraries identified several kinase inhibitors as HDT drugs for TB treatment, one of which has advanced into a clinical trial.^22–25^ However, it seems that no study has systematically investigated both the anti-TB and HDT potential of the early hits of ATP-competitive inhibitors.

Mtb glutamine synthetase (GS encoded by *glnA1*) was explored in our target-based screening for novel anti-TB leads. GS catalyzes the synthesis of l-glutamine from glutamate and ammonia in two steps: ATP phosphorylates the *γ*-carboxylate group of glutamate to form a *γ*-glutamyl phosphate intermediate; nucleophilic substitution of ammonia at the carbonyl group displaces the phosphate to give glutamine. Mtb GS is a chemically validated target.^26,27^ The *glnA1* gene essentiality has been demonstrated for in vitro and in vivo growth of Mtb, although its vulnerability was questioned.^28–30^ Aside from its pivotal role in nitrogen assimilation, an intriguing aspect of GlnA1 biology is that it is secreted, while a very low level of glutamine is present in the host cell. The extracellular GlnA1 activity has been associated with the pathogenicity (e.g., preventing phagosome acidification) and formation of a poly-l-glutamate/glutamine cell wall structure,^31^ strongly suggesting that GlnA1 is a virulence factor. Numerous high-throughput screening (HTS) studies identified diverse chemical scaffolds as GlnA1 inhibitors, one of which is the trisubstituted imidazole (compound 11a; Figure 1) with promising anti-TB activity.^32,33^ The HTS hits are mostly ATP-competitive inhibitors. Structural analysis has shown that the ATP-binding site and overall architecture of Mtb GlnA1 differ substantially from the human counterpart.^34^ Recently, a metabolomic study of Mtb found that the GlnA1 enzymatic activity is the most responsive to changes in ATP levels,^35^ sparking our interest in identifying new GlnA1 inhibitors that are active at the cellular level and could be used in a TB treatment regimen.

The biosynthetic activity of GlnA1 can be measured with a malachite green assay that detects the phosphate released during glutamine synthesis. The determined kinetic parameters of Mtb GlnA1 agree with the published literature values (Figure S1A–C).^26^ We optimized the assay into 20-*μ*L per reaction in 384-well format which had Z′ ~ 0.83 and signal-to-noise ratio ~4.8 (Figure S1D–E). We performed a miniature HTS using the Medicines for Malaria Venture (MMV) Pathogen Box that contains 400 diverse drug-like compounds.^36^ These compounds are active against neglected disease pathogens and at least 5-fold more selective for a pathogen than its host. As the hit rate of the initial screen that had 20 *μ*M compound per well was high (Figure 1A), 30 top hits were reassayed and the IC_50_’s were compared to the positive control 11a. The two screening results correlated reasonably well (Figure 1B). With resupplied compounds, four were confirmed with IC_50_ at ~1 *μ*M, but only MMV676605 was more potent than 11a (Figure S2). MMV676605 is the linsitinib (OSI-906, abbreviated as LIN) originally developed by OSI Pharmaceuticals. Commercial LIN (purity >98%) was included in a side-by-side comparison with 11a and two other GlnA1 inhibitors, l-methionine S-sulfoximine (MSO) and dl-phosphinothricin (PPT) (Figure 1C). PPT is a natural product-derived amino acid containing a phosphonic acid moiety. It mimics the GlnA1 reaction intermediate for the inhibition. MSO can be phosphorylated by GlnA1 at the imine group, resulting in a transition state analog tightly bound to the enzyme, almost irreversibly inhibiting GlnA1. As previously shown^37,38^ both MSO and PPT are glutamate-competitive; ATP can enhance the binding of the amino acid to GlnA1. By the IC_50_’s measured, LIN is the most potent biochemical inhibitor of Mtb GlnA1 (Figure 1D).

The inhibition mechanism of LIN was first analyzed using enzyme kinetics. The commercial EnzChek Phosphate Assay was adapted for continuously monitoring phosphate released during the reaction. A panel of reactions was set up with varying concentrations of one substrate (i.e., ATP for Figure 2A) and a fixed concentration of inhibitor while the other substrates were in excess. In the resulting Lineweaver–Burk plots (Figure 2A), 1/*V*_max_ is unchanged when ATP concentration increases, indicating LIN acts as an ATP-competitive inhibitor. In contrast, the LIN inhibition seems noncompetitive with regard to glutamate (Figure S3). Second, molecular modeling was used to predict LIN’s binding mode (Figure 2B–C) with the software AutoDock Vina.^39^ LIN was prepared and docked to the GlnA1 cocrystal structure 3ZXV with default parameters. From the docking results, the imidazopyrazine core is found to align well with the adenine of ATP (Figure 2D) and forms a *π*–*π* interaction with the benzene of residue Phe232. The amine group and its adjacent nitrogen of the core form three H-bonds with the hydroxyl of Ser280 and the main-chain carbonyl of Lys361. In addition, a H-bond forms between a hydroxyl on the cyclobutyl moiety of LIN and the *γ*-carbonyl of Glu214 of GlnA1. The imidazopyrazine and methylcyclobutanol substructures of LIN occupy essentially the same space as the adenosine of ATP. In a cocrystal structure ofthe GlnA1–11a complex (Figure S4), the pyridylamine, imidazole ring, and *tert*-butyl moieties of 11a occupy roughly the same space, but there are two H-bonds between the pyridylamine and Ser280’s hydroxyl group.

Based on the sequence alignment of bacterial GlnA1 homologues (Figure S5), Ser280 is a highly conserved residue, and Glu214 is also very conserved with the exception of one alanine. We then performed mutagenesis and purified three mutant proteins: E214A, E214Q, and S280A. Glu214 mutations affect the binding affinity of ATP less than Ser280 mutation, as the difference of *K*_m,ATP_ of E214A or E214Q versus the wide-type (WT) is small (Figure 2E). Alanine mutation at either place reduces the overall catalytic efficiency, as the *k*_cat_/*K*_m_ of E214A or S280A is smaller than that of the WT. Ser280 accounts more for the binding of LIN, indicated by the much larger disassociation constant *K*_d_ of S280A. While the present mutagenesis data only partially support the predicted binding mode, they fit into the notion that LIN binds to the ATP-binding site and is an ATP-competitive inhibitor of GlnA1.

The minimum concentration of LIN required to inhibit the growth culture of Mtb H37Rv is 25–50 *μ*M. A similar growth inhibition concentration (GIC) of LIN determined with the defined method is ~21 *μ*M (n = 4). To test whether LIN engages the GlnA1 target in Mtb, we created an inducible CRISPRi mutant of *glnA1* with avirulent Mtb H37Ra, in which *glnA1* expression can be repressed at the transcriptional level by the exogenous inducer anhydrotetracycline (ATc).^40^ Microbial growth of the mutant GDI-KD-9 could be efficiently suppressed by ATc (Figure S6A), genetically validating the *glnA1* essentiality that was previously demonstrated with a different approach.^28^ When ATc was present at 10 or 100 ng/mL in a growth culture, increased susceptibility of GDI-KD-9 to LIN as well as MSO and 11a was observed, compared to that without ATc (Figures 3 and S6B). Importantly, the effect was specific to GlnA1 inhibitors including LIN because under similar conditions GDI-KD-9 remained susceptible to RIF, which targets RNA polymerase (Figure S6C). It is noted that the 16-fold GIC shift of MSO, caused by the reduction of *glnA1* expression, was much larger than the 3- to 4-fold GIC shift of LIN or 11a. MSO at high concentrations inhibits the growth completely while the inhibition of LIN or 11a saturates at 20% full scale (Figures 3 and S6B). The difference could be attributed to the almost irreversible inhibition mechanism of MSO. It also implies that the in vitro bactericidality or solubility of LIN and 11a is poor. Synthetic modification is needed to improve LIN’s anti-TB activity.

LIN is an anticancer drug that has been investigated in multiple clinical trials. The cytotoxicity (CC_50_) of LIN was measured at 67.5 *μ*M against Vero E6 cells, compared to the much higher toxicity reported against abnormal tumor cells.^41^ Even so, LIN did not inhibit recombinant human GS in a biochemical reaction while 11a did inhibit it considerably (Figure S7). LIN is a rationally designed and optimized inhibitor of insulin-like growth factor 1 receptor and insulin receptor (IGF1R/IR) belonging to the tyrosine kinase family.^42^ The exquisite selectivity of LIN has been demonstrated by testing on 442 human kinases.^43^ LIN only inhibits IGF1R/IR kinase activity in vitro and in mammalian cells. The inhibition mechanism was illustrated with a cocrystal structure, where LIN interacts with the key ATP-binding residues of IGF1R.^42^ LIN could exert its cellular function through the IGF1R-mediated signaling cascade that progresses through the PI3K/AKT and Raf/MEK/ERK pathways. Stimulating these pathways by IGF1R/IR activation ultimately leads to cell proliferation and survival. Inhibition of IGF1R/IR activation including its autophosphorylation would have the opposite effect, resulting in autophagy or cell death. LIN was one of seven classes of IGF1R/IR inhibitors disclosed before 2012.^43^ When LIN’s intramacrophage anti-TB activity was to be examined, Av-Gay and co-workers published the results of a high-content screening for kinase inhibition that could enable intracellular killing of Mtb.^25^ LIN was one of the kinase inhibitors included in the screening. In that study, IGF1R was ranked the second highest target for anti-TB HDTs, but LIN’s host-targeting activity had not yet been confirmed and the response of Mtb-infected macrophages upon LIN treatment was not analyzed. More recently, the HDT potential of LIN was demonstrated as an antiviral strategy to counteract SARSCoV-2 infection.^44^ An anticancer co-immunotherapy study also showed IGF1R inhibition by LIN could activate autophagy.^45^

We first tried to confirm the autophagy-activating ability of LIN using human macrophage THP1 cells. As shown in Figure 4A, LIN treatment induced autophagy in THP1 macrophages, as increased lipid bound LC3B-II converted from LC3B-I was observed in comparison to that of untreated control (Figure 4A). No difference in the level of cleaved caspase 3 was found, indicative of no apoptosis induction (Figure 4A) and no consequential cell death. Moreover, the increased autophagy flux was found in the uninfected (UI) or Mtb-infected THP1 macrophages as the level of p62 was seemingly reduced after preincubation with LIN (Figure 4B). To further confirm LIN-induced autophagy activation, mRFP-GFP-LC3 reporter THP-1 cells were used to show LC3 enrichment in autophagosomes and autolysosomes by confocal microscopy with rapamycin as a positive control.^46,47^ The result demonstrated that the autophagosome and autolysosome forming puncta were elevated in both Mtb-infected and uninfected THP1 macrophages after LIN treatment (Figure 4C–E). The mTOR targeted by rapamycin is closely associated with autophagy; its phosphorylation inhibits autophagy induction.^48^ To investigate the relationship between LIN-triggered autophagy and mTOR activity, LIN treated THP1 macrophages were subjected to Western blotting analysis. We found that LIN can reduce the level of mTOR phosphorylation in both uninfected and Mtbinfected macrophages (Figure 4B), in agreement with autophagy activation. These results indicate that LIN improves autophagy flux through mTOR signaling, but how the mTOR pathway is affected by the LIN inhibition of IGR1F/IR remains unknown. Also, the LIN concentration tested is higher than the IC_50_ needed to inhibit IGF1R/IR in cancer cells; other cellular mechanisms may be involved in the autophagy activation.

A number of studies have shown that autophagy occurrence contributes to the elimination of intracellular bacteria such as Mtb.^2^ Here, it was hypothesized that LIN-mediated autophagy might lead to controlling intracellular Mtb although the in vitro antibacterial activity of LIN measured by the minimum inhibition concentration seems not very strong. As expected, LIN treatment significantly decreased the survival of Mtb H37Rv within macrophages in a dose-dependent manner (Figure 5A). It is noted that even at 5 or 10 *μ*M, a concentration that is far less than the GIC, LIN alone was still capable of reducing the bacterial counts. Similar results were observed in the macrophages infected with a clinically isolated isoniazid-resistant strain of Mtb (Figure 5B). Furthermore, when combined with the TB drug bedaquiline, which has a delayed killing mechanism to treat H37Rv-infected THP1, LIN eliminated more intracellular Mtb and improved the killing effect of BDQ in 72 h to a significant level (Figure 5C). The preliminary results suggest that LIN could be explored further for HDT using animal infection models.

In summary, through target-based biochemical screening, we identified a human kinase inhibitor drug LIN as a potent inhibitor of Mtb essential enzyme, GlnA1. LIN’s mechanism of inhibition against GlnA1 was studied with enzyme kinetics, protein mutagenesis, and molecular modeling methods. LIN is shown as an ATP-competitive inhibitor of GlnA1, with the same mode of action it employs to inhibit human kinase IGF1R/IR. LIN is a direct antimycobacterial at ~25 *μ*M or above, which is a result of inhibiting GlnA1 in Mtb. We further analyzed the cellular response and consequences of LIN treating Mtb-infected THP1 macrophages. LIN was found to be able to activate autophagy in the macrophage and kill both drug-sensitive and resistant Mtb in THP1 cells. Therefore, LIN joins a growing list of recently identified kinase inhibitors that can be repurposed for antimicrobial chemotherapy to treat TB or other bacterial or fungal infections.^49–51^ Although LIN has excellent drug-like properties in terms of metabolism and pharmacokinetics,^42^ its antibacterial potency as an early hit must be improved, particularly with concerns about its solubility and permeability into Mtb to inhibit GlnA1. Future optimization would take advantage of the Mtb GlnA1 and human IGF1R/IR protein crystal structures. The ATP binding site of IGF1R/IR accommodates at least 10 diverse chemical scaffolds^43^ (references herein), one of which is a pyrrolotriazine named BMS-754807, which was also identified as a top hit against Mtb phenylalanyl-tRNA synthetase (PheRS) in our previous screening.^52^ PheRS is an ATP-dependent enzyme. It is tempting to ask whether other IGF1R/IR inhibitors and their analogues can inhibit bacterial ATP-dependent enzymes. Nevertheless, inhibiting IGF1R/IR is perhaps an effective way of HDT to control microbial growth in infectious diseases.

## METHODS

### Microbiological Experiments and Chemicals.

The wild-type strains used are Mtb H37Rv ATCC27294 for GIC and H37Ra ATCC25177 for genetics. CRISPR plasmid pLJR965 was from Addgene (#115163). Mtb was cultured in 7H9 complete medium which is Middlebrook 7H9 medium base (BD-Difco) supplemented with 10% oleic acid-albumin-dextrose-catalase (OADC, BD-BBL), 0.2% glycerol, and 0.05% tyloxapol (Sigma). Cultures were incubated at 37 °C in covered culture flasks with or without agitation. The mycobacteria were plated onto Middlebrook 7H10 agar plates with 7H10 base supplemented with 10% OADC and 0.5% glycerol and incubated at 37 °C for 3–4 weeks. Commercial linsitinib was from MedChemExpress. PPT and MSO were from Sigma. Compound 11a was provided by TB Alliance.

### HTS and Kinetic Assays.

The HTS assay measures the phosphate released at an end-point of the GlnA1 reaction using malachite green reagent (BIOMOL, Enzo). The screening was carried out in 384-well microplates (Corning #3764). The reaction buffer contains 50 mM HEPES pH 7.4, 10 mM MgCl_2_, and 0.01% Brij-35 (Sigma). Library compounds were prepared in the assay plate using Echo for a final concentration of 20 *μ*M. DMSO was used as the negative control, and 10 *μ*M tool compound 11a as the positive control. Ten *μ*L of reaction buffer containing GlnA1 for the final 10 nM was added and incubated with the compound in a well for 30 min. Then 10 *μ*L of reaction buffer containing substrates (0.8 mM ATP, 20 mM l-Glu, and 40 mM NH_4_Cl at final concentration) was added to start the reaction at 25 °C, and the reaction stood for 2 h. Finally 30 *μ*L of malachite green reagent was added. The plate was incubated for 5 min before 10 *μ*L of sodium citrate was added at 150 mM and incubated for 30 min. The absorbance at 620 nm was recorded using Envision (PerkinElmer). Kinetic analysis of phosphate production used a continuous assay adopted from the EnzChek Phosphate assay kit (Thermo) with little modification. It was performed in the 384-well microplate with the same components and concentrations in the reaction buffer as above. The 20 *μ*L reaction included 5 or 10 nM GlnA1 or human GS, a series concentration of substrates, 0.05 mM MESG, and 0.1 unit/mL PNPase from the kit. Absorbance at 360 nm was read every minute for 1–2 h.

### CRISPRi Constructs and GIC Shift Experiment.

The standard protocol of CRISPR interference for programmable transcriptional repression in mycobacteria was followed.^40^ We searched the *glnA1* (Rv2220) genetic locus with the highest-scoring PAM 5′-NNAGAAG and identified the sequence 5′-AGGGTGAACGGGTCGTGCAC for sgRNA that can base-pair with the coding strand of *glnA1*. This sequence was appended with GGGA nucleotides at the 5′-end (for cloning) and annealed with a complementary DNA oligo. The double-stranded DNA was ligated into vector pLJR965 that was digested with BsmBI-V2 (NEB), to yield plasmid pKD-9. pKD-9 was transformed to *E. coli* DH5*α* and selected by 100 *μ*g/mL kanamycin resistance. The confirmed plasmid was transformed into H37Ra and selected on 7H10 plates with 25 *μ*g/mL kanamycin. The PCR-confirmed *glnA1* knockdown mutant is named GDI-KD-9. A few GDI-KD-9 colonies were cultured in 7H9 complete medium until OD_600_ ~ 0.6. The culture was diluted to a starting inoculation OD_600_ of 0.01 and grown in 96-well plates in the presence of either 0 ng/mL, 10 ng/mL, or 100 ng/mL ATc. LIN and MSO were set up in a 9-point, 2-fold dilution gradient with concentrations starting from 150 *μ*M and 100 *μ*M, respectively, in a total volume of 200 *μ*L/well. After exposure to inhibitors and inducer for 7 days at 37 °C, each well was added with 50 *μ*L of reagent that is a mixture (v/v 1:1) of alamarBlue (Thermo) and 10% Tween 80 and incubated for another 20 h. The minimum inhibitory concentration was determined by the concentration of compound that turned the wells blue. Then, the fluorescence of the wells with excitation and emission respectively at 544 and 590 nm was recorded using BMG OMEGA. Using DMSO treated wells as control, the processed data were plotted as the logarithm of concentration versus the percentage of inhibition and fit into a nonlinear regression model that is the “Find ECanything” function in the GraphPad Prism. With F constant set to 90, GIC was calculated.

### Cell Culture.

The human monocytic cell line (THP-1) and THP-1 cells harboring an mRFP-GFP-LC3B reporter (kindly provided by Professor Xinchun Chen, Shenzhen University, Shenzhen, China) were cultivated in RPMI 1640 (Gibco) culture medium containing 10% fetal bovine serum (FBS; Invitrogen, Life Technologies), 1% HEPES (Gibco), and 1% sodium pyruvate (Gibco) at 37 °C and 5% CO_2_. THP-1 macrophages were differentiated as previously described.^46,47^

### Colony Forming Unit (CFU) Counting.

PMA-differentiated THP-1 macrophages (2.5 × 10^5^ cells) pretreated with LIN (10 *μ*M) for 1 h were infected with H37Rv or a clinical INH resistant Mtb at a multiplicity of infection (MOI) of 10 for 4 h at 37 °C, 5% CO_2_. The macrophages were washed and then treated with LIN for another 24, 48, or 72 h. Cells were lysed and then diluted for CFU counting.

### Confocal Microscopy.

PMA-differentiated and mRFP-GFP-LC3B expressed THP-1 cells were preincubated with LIN (10 *μ*M) for 1 h and then infected with Mtb H37Rv (MOI = 10) for 4 h. After washing with PBS, the macrophages were treated with LIN for 24 h. The infected cells were fixed, stained, and then observed and photographed using an Olympus FV1000 confocal microscope (Nikon A1R).

## Supplementary Material

Supporting information

## Figures and Tables

**Figure 1.**
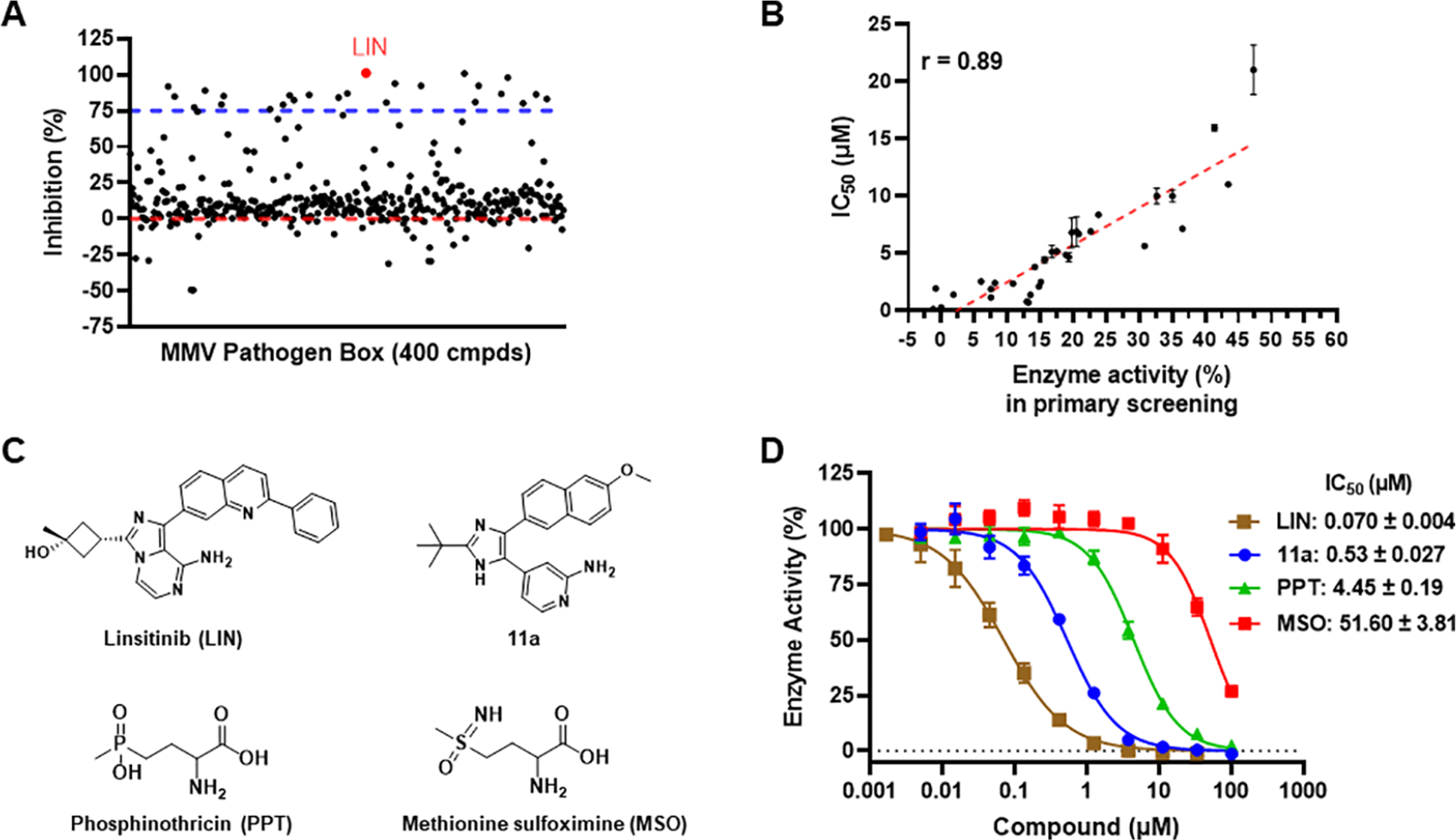
Results of screening Mtb GlnA1 with the MMV Pathogen Box library and hit confirmation. (A) Summary plot of the primary screening. The hit linsitinib (LIN) is marked in red. Other top hits are shown in Figure S2A. (B) Correlation analysis between the data of the initial primary screening assay and the dose–response retest for select hits. (C) Structures of LIN, 11a, PPT, and MSO. (D) Dose–response curves of the compounds and the IC_50_ values (drug concentration that produces a 50% maximal inhibition) against Mtb GlnA1 measured by a malachite green assay, presented as mean ± SE (*n* = 3).

**Figure 2.**
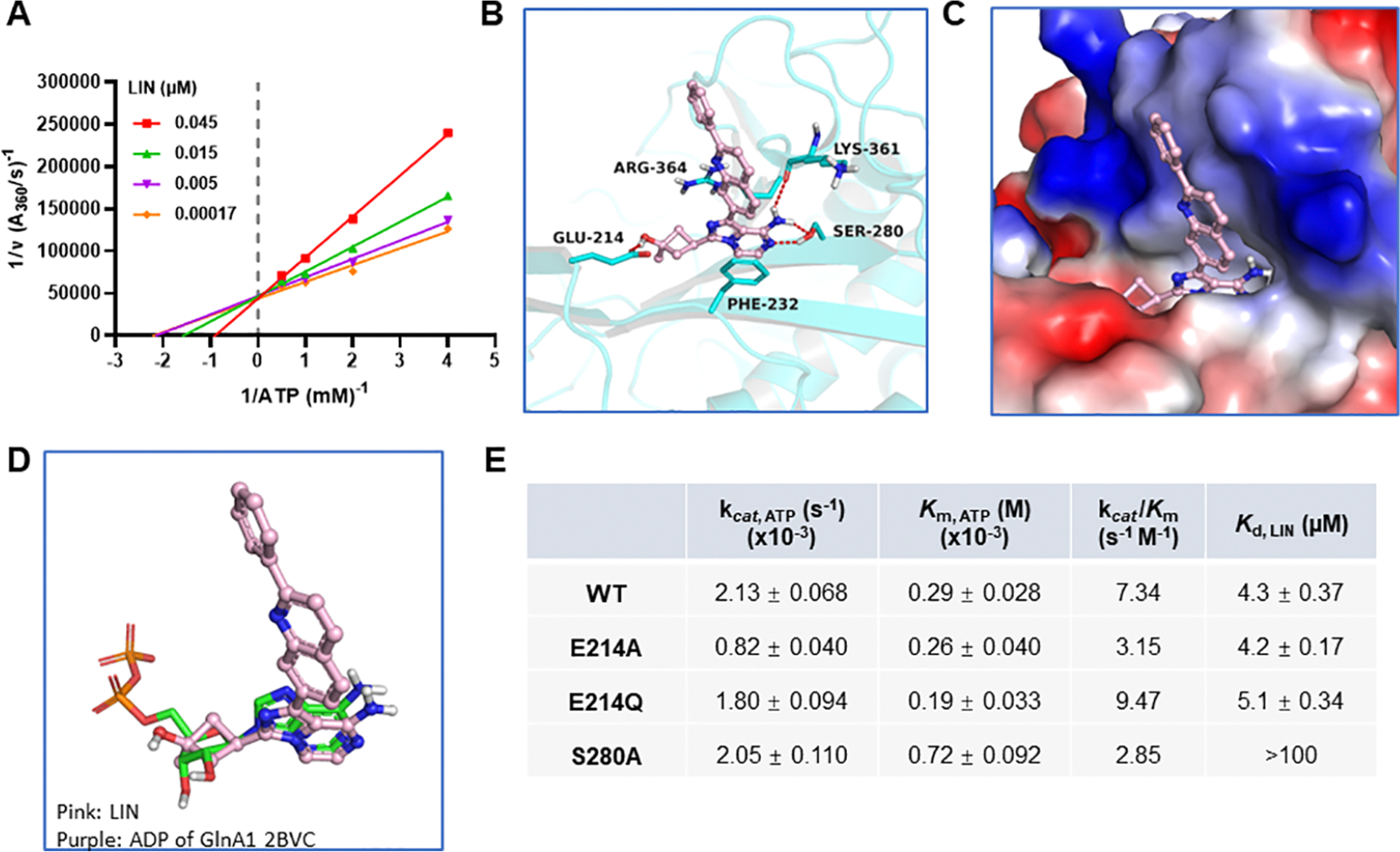
Mechanism of inhibition of LIN. (A) Lineweaver–Burk plot showing the competitive inhibition mode with regard to ATP. *K*_*i*_ = 0.024 ± 0.0020 *μ*M. (B) Proposed binding mode of LIN against Mtb GlnA1. LIN is represented in pink. The Mtb GlnA1–11a cocrystal structure (PDB: 3ZXV) is shown in cyan with the major interaction residues labeled. (C) Electrostatic potential map of Mtb GlnA1 with LIN. (D) Overlapping the pose of LIN (pink) in the model with adenosine diphosphate ligand (green) in the GlnA1–ADP cocrystal structure (PDB: 2BVC). (E) Comparison of the steady-state kinetic values of *k*_cat_ and *K*_m_ with regard to ATP and the disassociation constants *K*_d_ of LIN for GlnA1 wild-type (WT) and the three mutants. See the Methods section.

**Figure 3.**
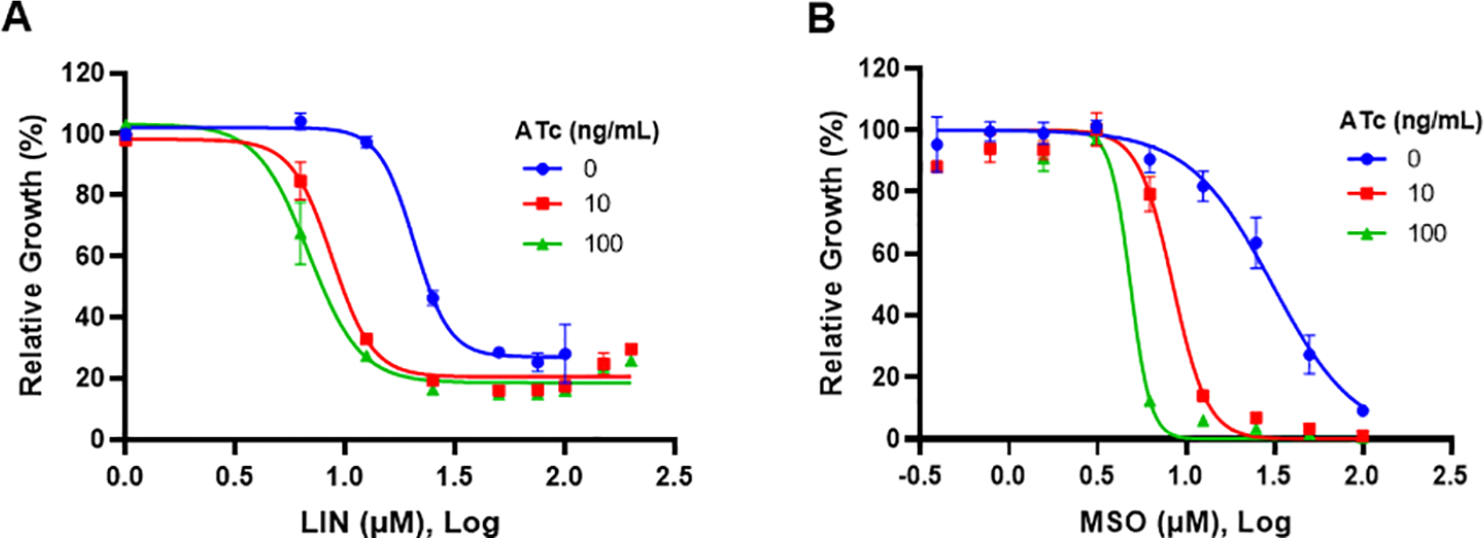
Transcriptional silencing of *glnA1* by inducible CRISPRi hypersensitizes Mtb to *glnA1* inhibitors. GICs were tested for the susceptibility of GDI-KD-9, the Mtb H37Ra-derived *glnA1* knockdown mutant, to (A) LIN and (B) MSO under ATc inducing or noninducing conditions as indicated. Each data point represents the average and standard error of three technical replicates in a representative experiment. The data set was fitted into a nonlinear regression model. The experiment for each compound was repeated at least once.

**Figure 4.**
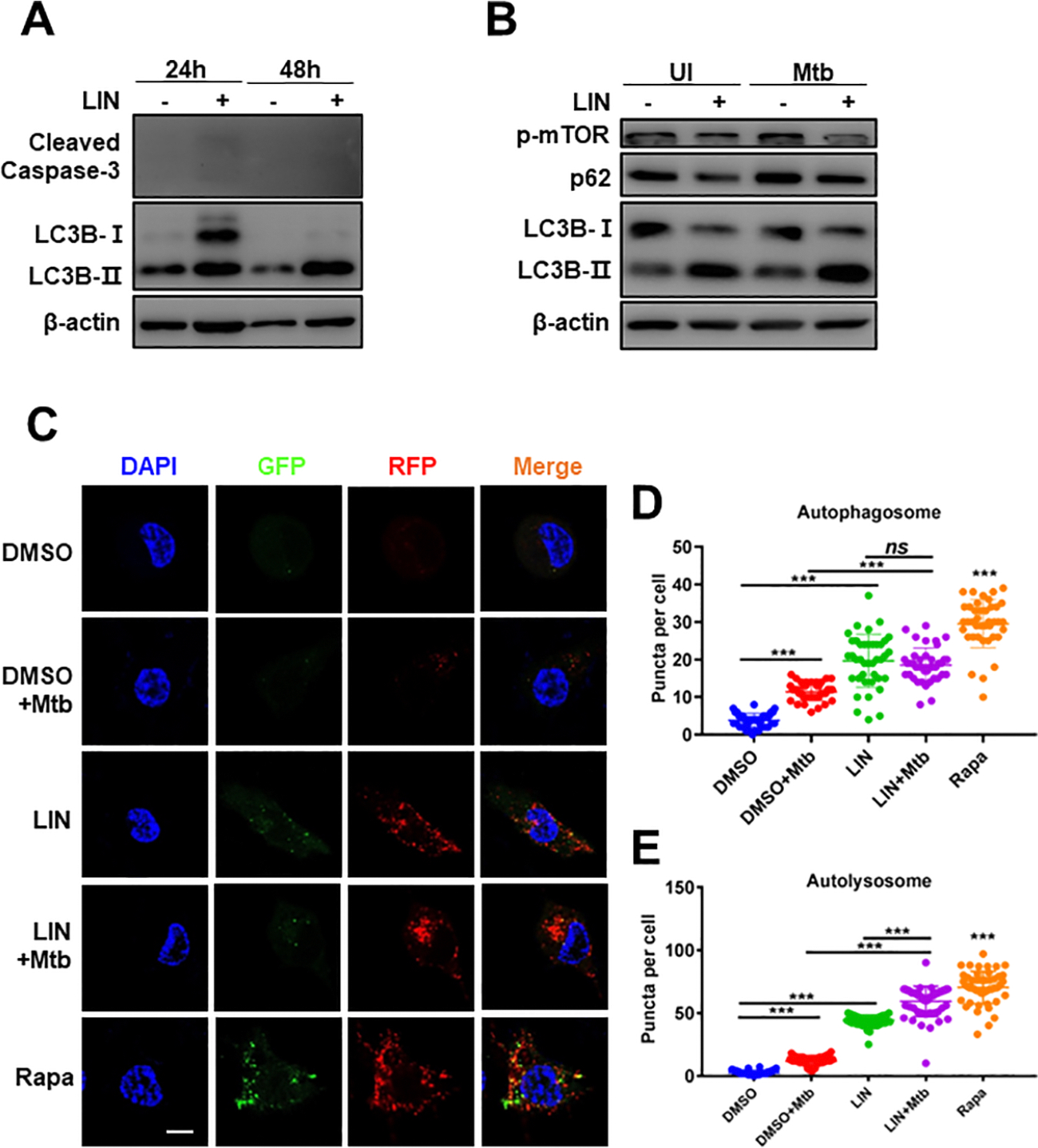
LIN treatment enhances autophagy flux in THP-1 macrophages. (A) THP-1 macrophages were incubated with 10 *μ*M LIN for the indicated times, and then the cell lysates were subjected to Western blotting analysis using the indicated antibodies. *β*-Actin was used as the loading control. (B) THP-1 macrophages were pretreated with 10 *μ*M LIN for 1 h followed by Mtb H37Rv infection (MOI = 10:1) or no infection (UI) for 24 h. The cell lysates were subjected to Western blotting analysis using the indicated antibodies. *β*-Actin was used as the loading control. (C) mRFP-GFP-LC3B reporter THP-1 macrophages were infected with Mtb H37Rv (MOI = 10:1) in the presence or absence of 10 *μ*M LIN for 24 h and then analyzed by confocal microscopy. Results are shown as one of representative images (bar, 5 *μ*m). The autophagosome puncta (yellow) per cell (D) and the autolysosome puncta (red) per cell (E) were calculated. The results are shown as the means ± standard deviations (SD), *n* = 3. ***P < 0.001; *ns*, not significant. Also see refs 46 and 47.

**Figure 5.**
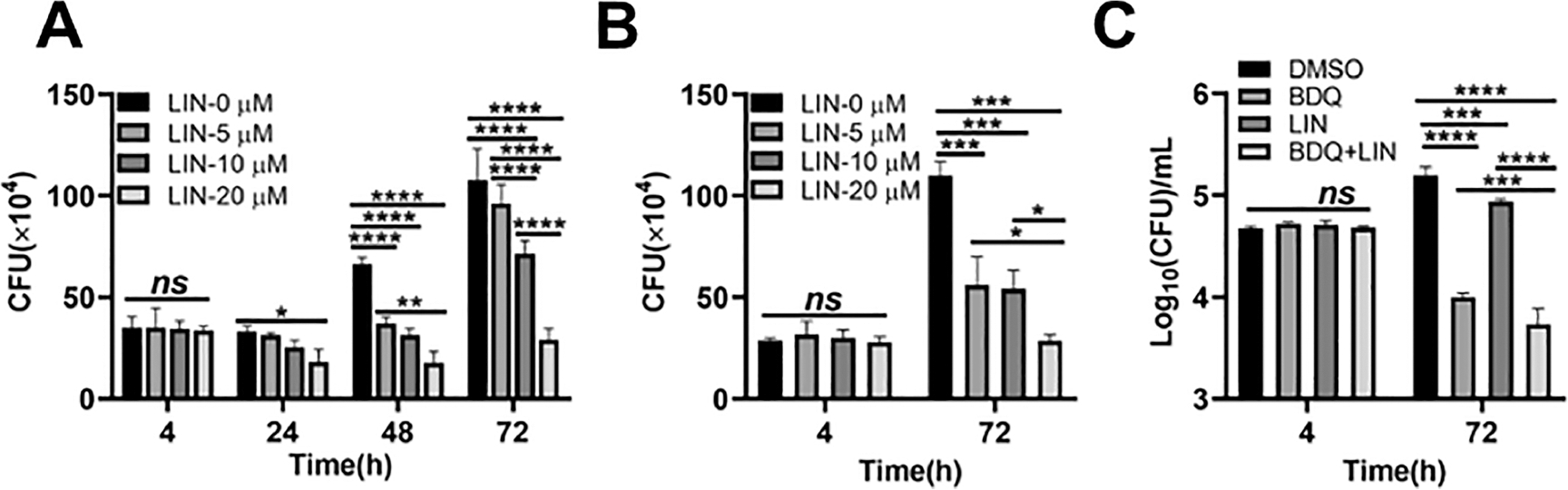
LIN-mediated autophagy inhibits intracellular Mtb survival within THP-1 macrophages. THP-1 macrophages preincubated with LIN for 1 h were infected with Mtb H37Rv (A) or a clinical INH-resistant Mtb strain (B) (MOI = 10:1) for 4 h. After removing extracellular bacteria by washing with warm PBS, the infected THP-1 macrophages were incubated with the indicated concentrations of LIN for another 24, 48, and 72 h. Cells were lysed, and CFU counting was performed. (C) THP-1 macrophages preincubated with LIN for 1 h were infected with H37Rv (MOI = 10:1) for 4 h. After removing extracellular bacteria by washing with warm PBS, the infected THP-1 macrophages were incubated with 10 *μ*M LIN in combination with 0.2 *μ*g/mL bedaquiline (BDQ) for another 72 h. Cells were lysed, and CFU counting was performed. The results are shown as the means ± standard deviations (SD), *n* = 3. *P < 0.05; **P < 0.01; ***P < 0.001; ****P < 0.0001.
