# Supplementary material for: Human Kinase IGF1R/IR Inhibitor Linsitinib Controls the In Vitro and Intracellular Growth of Mycobacterium tuberculosis
Source: ACS Infect Dis. Author manuscript; Available in PMC 2025 Feb 9. (PMC11807261; doi:10.1021/acsinfecdis.2c00278)
Supplement: Supporting information [file NIHMS2048699-supplement-Supporting_information.pdf]

# Supporting Information

## Human kinase IGF1R/IR inhibitor Linsitinib controls the in vitro and intracellular growth of *Mycobacterium tuberculosis*

Heng Wang[1†], Jing Bi[2†*], Yuan Zhang[1], Miaomiao Pan[1], Qinglong Guo[2], Genhui Xiao[1], Yumeng Cui[1], Song Hu[1], Chi Kin Chan[1], Ying Yuan[1], Takushi Kaneko[3], Guoliang Zhang[2], and Shawn Chen[1*]

[1]Global Health Drug Discovery Institute, Haidian, Beijing 100192, China

[2]National Clinical Research Center for Infectious Diseases, Guangdong Provincial Clinical Research Center for Tuberculosis, Shenzhen Third People's Hospital, Southern University of Science and Technology, Shenzhen 518112, China

[3]Global Alliance for TB Drug Development, New York, NY 10005, USA

[†]These authors contributed equally to this work.

*Co-corresponding authors: Jing Bi (11210700007@fudan.edu.cn) and Shawn Chen (shuo.chen@ghddi.org)

## ADDITIONAL EXPERIMENTAL DETAILS, MATERIALS AND METHODS

**GlnA1 plasmid construction.** The native sequence of Mtb glnA1 (NCBI gene ID: 888383) was cloned into the pET28 expression vector, which would result in a GlnA1 protein fused with N-terminal His6 tag. Three mutated GS expression plasmids were made by using the Fast Mutagenesis System (Transgen) and DNA oligos with mutational nucleotides that lead to one amino acid change in the designated residue of a mutant protein of GlnA (E214A, E214Q or S280A).

**GlnA1 protein expression and purification.** The GlnA1 expression plasmids were transformed to BL21(DE3) competent cells. A single colony was inoculated into a 20 mL LB medium containing 20 μg/mL kanamycin and incubated overnight at 37 °C. A fresh culture of 10 ml was used to inoculate 1 L medium and grown at 37 °C until $OD_{600}$ reached ~1.0. The culture was cooled down to 20 °C, and then 0.5 mM IPTG was added. After 18 h of incubation at 16°C, cells were harvested by using centrifuge (3800g, 15 min, 4 °C) and resuspended with cell harvesting buffer (25 mM Tris pH 8.0, 150 mM NaCl, 10 mM imidazole pH 8.0, 10% glycerol, 1 mM DTT, and EDTA-free protease inhibitor tablet [Roche]). Resuspended cells were broken open by ultrasonication. Cell debris was removed through centrifugation (24,000g, 80 min) followed by filtration against 0.22 μm polyvinylidene fluoride membrane (Millipore), and then add 2 mL Ni-NTA agarose resin (Thermo) to 40 mL lysates. After incubation with soft shaking, the mixtures were run three times washing with washing buffer (25 mM Tris pH 8.0, 150 mM NaCl, 20 mM imidazole pH 8.0, 10% glycerol, and 1 mM DTT). Eluted protein with 4ml elution buffer (25 mM Tris pH 8.0, 150 mM NaCl, 200 mM imidazole pH 8.0, 10% glycerol, and 1 mM DTT). Proteins were concentrated and changed buffer (50mM HEPES pH7.4, 150mM NaCl, 10% glycerol) by Amicon Ultra-4 device (Millipore). The purity which is very often >98% by this method and concentration were determined by SDS-PAGE and BCA kit (Thermo), then dispensed into 20 μL aliquots and stored at -80 °C.

**Protein thermal shift assay.** SYPRO Orange (Thermo) was used as the fluorescent dye in the assay. The reaction buffer was the same as above. A 10 μL mixture containing 2 μM GlnA1 and 10x SYPRO Orange dye was added to each well of MicroAMP EnduraPlate 384-well qPCR plate (Thermo). The running progress was performed on the Q7 Flex qPCR instrument (ABI). Protein Tm was calculated by using Protein Thermal Shift software (Thermo), and the derivative Tm (TmD) was taken as protein Tm. For determining $K_d$ of LIN binding to GlnA1 or a mutant, a series of 12 concentrations of compound was transferred into qPCR plate by Echo. $K_d$ was calculated by plotting TmD against LIN concentration and fit to an equation (J. Vis. Exp. 91:51809).

**Growth curve measurement.** Mtb H37Ra WT and GDI-KD-9 strains were cultured till log phase (OD600 ~0.5) in the Middlebrook 7H9 medium base supplemented OADC, 0.2% glycerol and 0.05% Tween-80 (Sigma). The cultures were diluted to OD600 ~0.001 separately with 25-ml and 100-ml 7H9 complete medium. The diluted GDI-KD-9 culture was aliquoted to 4 flasks with 25 ml each, and ATc (Anhydrotetracycline) was added to final concentrations at 0ng/ml, 10ng/ml, 50ng/ml and 100ng/ml respectively. These were cultured at 37℃ with 100rpm. Optical density at OD600 was measured with a spectrometer at different time points and the data was used to plot growth curves with GraphPad Prism.

**Western blot analysis.** PMA-differentiated THP-1 macrophages ($5 \times 10^5$ cells/mL) treated with LIN (10 μM) with or without Mtb H37Rv infection (MOI = 10) were subjected to Western blotting analysis. Briefly, the membrane blocked with 5% nonfat milk was incubated with antibodies against LC3B (Sigma), actin (Cell Signaling Technology), p62 (Abcam), Cleaved-caspase 3 (Cell Signaling Technology), and p-mTOR (Cell Signaling Technology) at 4°C overnight. After washing and incubation with corresponding second antibody (Cell Signaling Technology), the membrane was exposed using enhanced chemiluminescence (ECL) detection solution (Thermo).

**Data analysis.** GraphPad Prism 9.10 was used for data analysis. Compound dilutions were prepared using Echo in a series of 10 concentrations. The remaining enzyme activity was calculated:

$$\text{Activity (\%)} = \left(\frac{\text{Abs}^{I} - \text{Abs}^{max}}{\text{Abs}^{max} - \text{Abs}^{min}}\right) \times 100$$

$\text{Abs}^{I}$ was the reading from an inhibitor well, $\text{Abs}^{max}$ was from DMSO control well, and $\text{Abs}^{min}$ was from the tool compound well. The activity percentage was plotted against inhibitor concentration. To calculate $IC_{50}$, the resulting dose-response curve was fit to Equation 1, where H was the Hill slope factor.

$$y = y_{min} + \frac{y_{max} - y_{min}}{1+\left(\frac{IC_{50}}{x}\right)^{H}} \qquad \text{(Equation 1)}$$

For analysis of the mode of inhibition with the kinetic assay data, Lineweaver–Burk plots were fitted for modeling of inhibition. To calculate the Ki for competitive inhibition, the plots with Pi production rate against substrate concentrations were fit to:

$$v_0 = \frac{\text{V}_{max}[\text{S}]}{K_M\left(1+\frac{[I]}{K_i}\right)+[S]} \qquad \text{(Equation 2)}$$

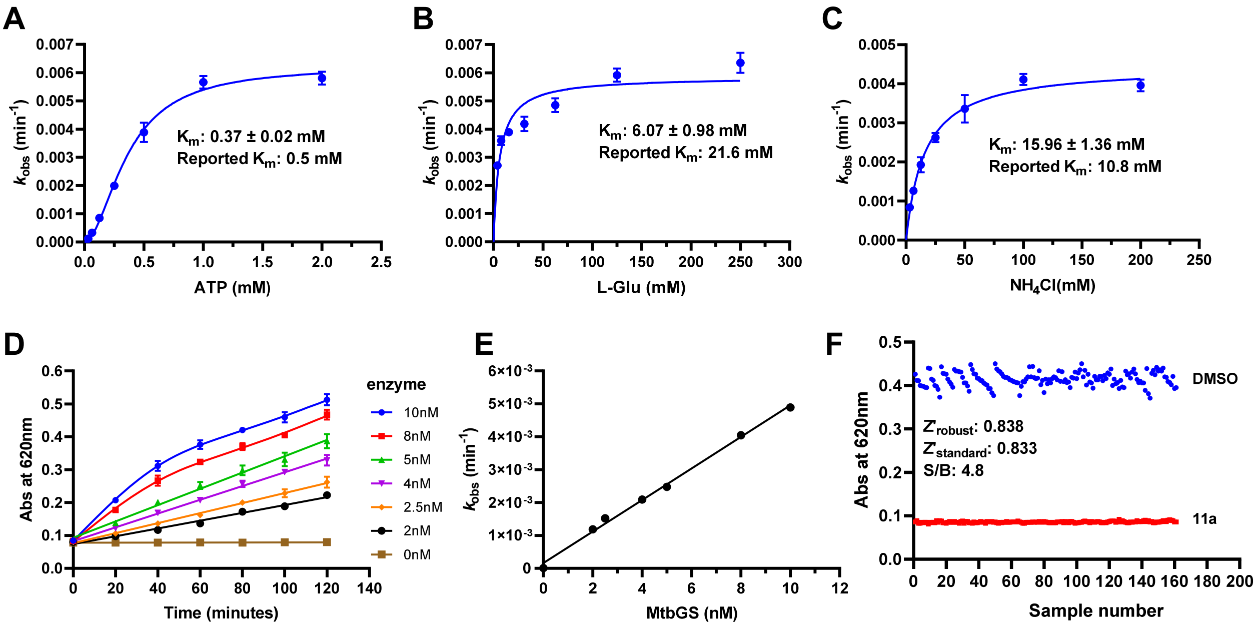


**Figure S1.** Development of HTS screening for GlnA1 inhibitor. (A-C) $K_m$ determination of Mtb GlnA1 with respect to ATP, L-Glu and $NH_4^+$, $K_m$ values in this assay are marked in black, and reported $K_m$ parameters are marked in bold letters. (D) Reaction rates at different concentrations of GlnA1. (E) linearity of reaction rate with respect to Mtb GlnA1 concentration. F, Z' factor of HTS by using malachite green assay.

**A**

| | MMV676605 (Linsitinib) | MMV011903 | MMV659004 | MMV030734 | MMV021013 |
|---|---|---|---|---|---|
| **Structure** | 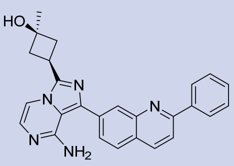 | 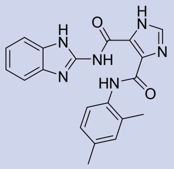 | 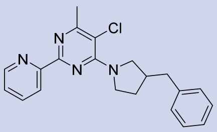 | 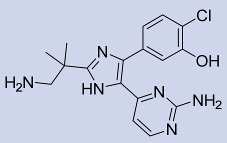 | 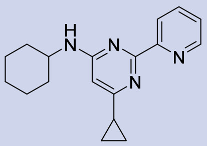 |
| **$IC_{50}$ (μM), library** | 0.13 | 0.78 | 1.14 | 1.38 | 0.69 |
| **$IC_{50}$ (μM), resupply** | 0.070 ± 0.004 | 1.63 ± 0.20 | 0.66 ± 0.04 | 1.84 ± 0.09 | 0.69 ± 0.08 |

**B**

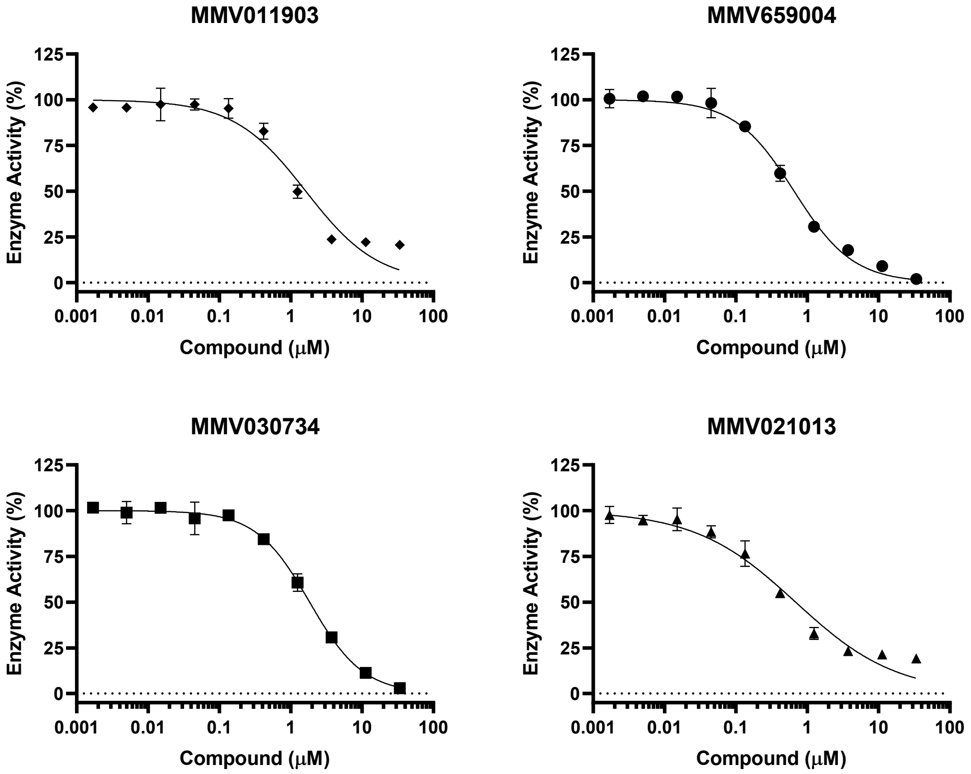


**Figure S2**. Top hits of Mtb GlnA1 HTS and the $IC_{50}$'s determined. (A) Structures of the top hits. The first row of $IC_{50}$'s was determined with compounds directly from MMV Pathogen Box library plates. The second row of $IC_{50}$'s was determined with compounds resupplied from MMV. Error is presented as mean ± SE (n=3). (B) The dose-response curves of $IC_{50}$'s determined with the resupplied compounds. The dose-response curve of MMV6766505 is shown in Figure 1D.

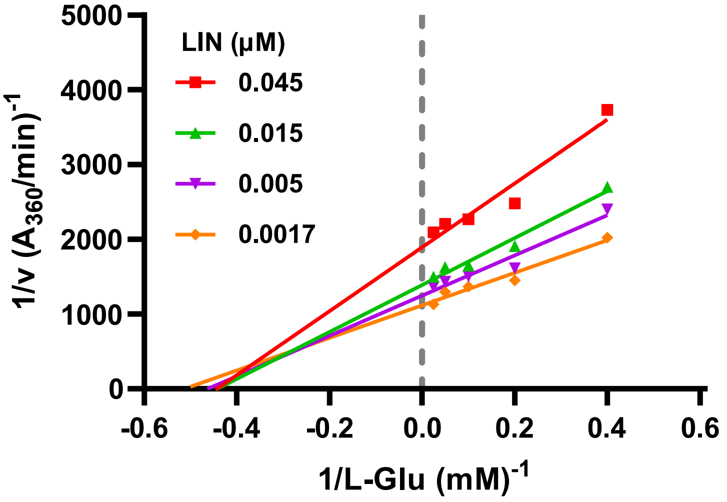


**Figure S3.** Mode of inhibition of LIN against Mtb GlnA1, analyzed by Lineweaver-Burk plot with respect to L-Glu.

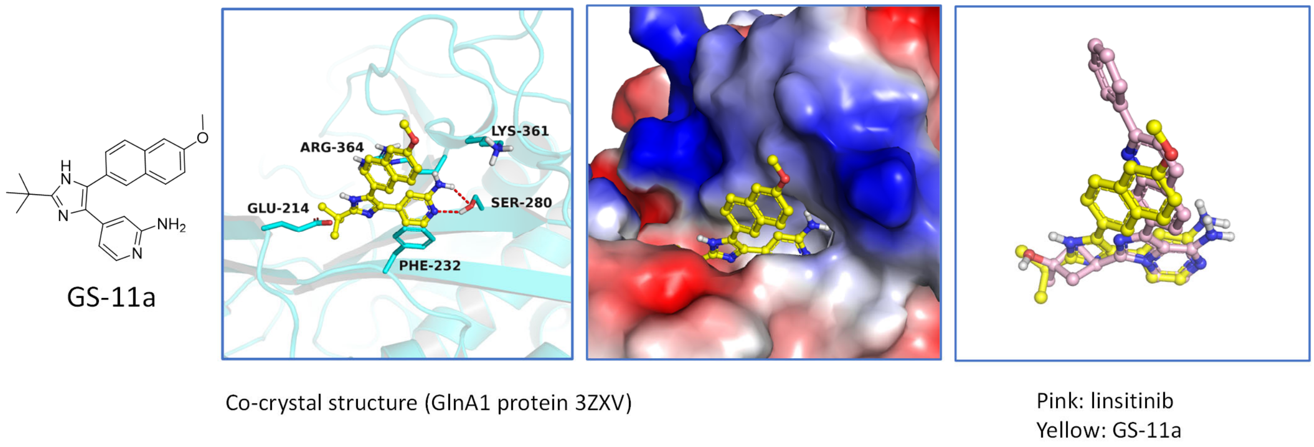


**Figure S4**. Binding representation of GlnA1 and 11a in cocrystal structure 3ZXV and alignment of LIN and compound 11a in the binding.

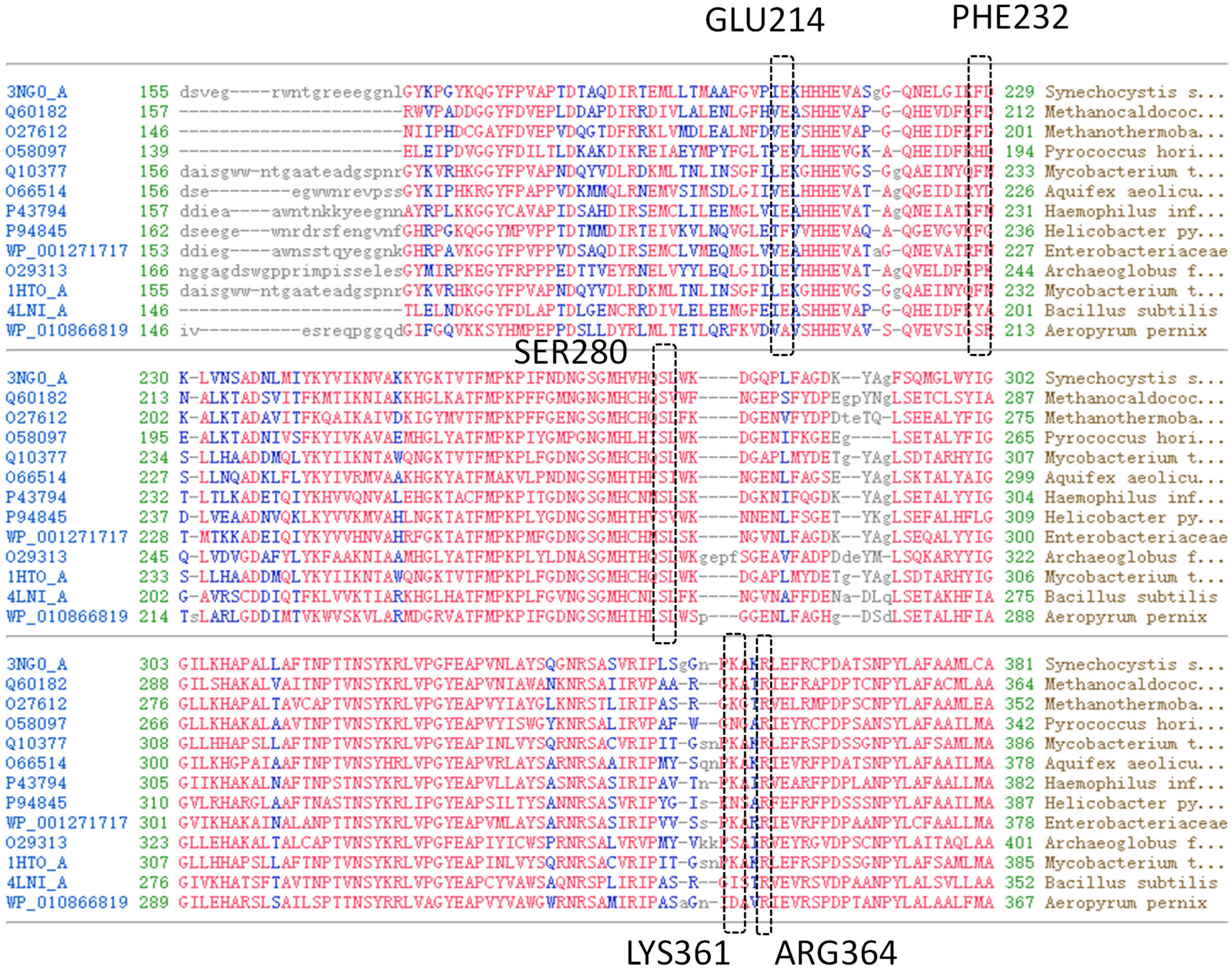


**Figure S5.** Conserved protein domain of GlnA1 provided by NCBI webserver. Source: https://www.ncbi.nlm.nih.gov/Structure/cdd/cddsrv.cgi?uid=273198

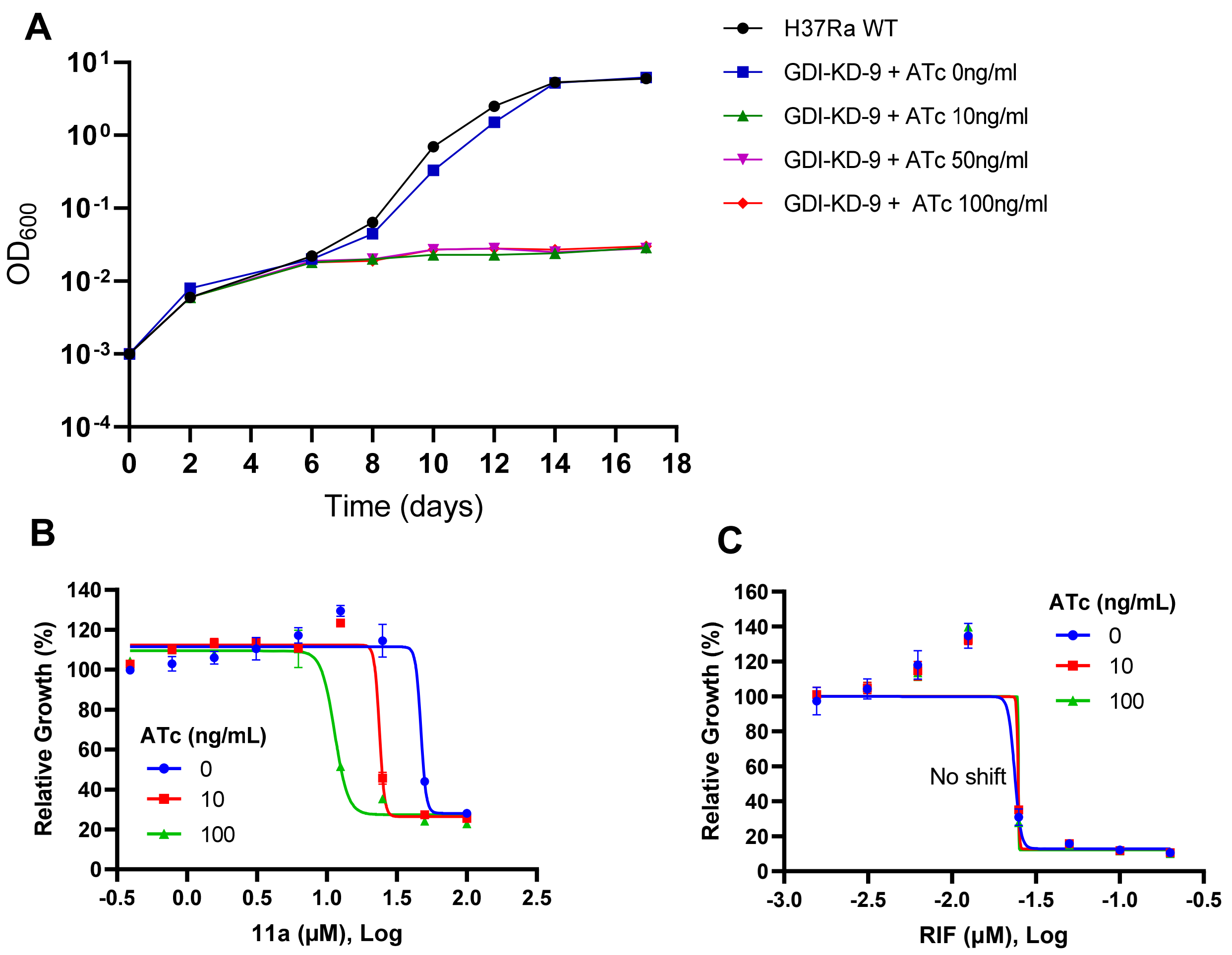


**Figure S6.** Transcriptional silencing of *glnA1* in *Mtb* H37Ra GDI-KD-9 strain by CRISPRi increased the susceptibility to GlnA1 inhibitor 11a and showed no effect on the susceptibility to rifampicin (RIF) that targets RpoB. (A) Growth curves of the mutant strain GDI-KD-9 in the presence of varying concentrations of inducer ATc, compared to wild-type H37Ra. (B) GDI-KD-9 was 3.6 times more sensitive to 11a when cultured in the presence of 100 ng/ml ATc. C) GDI-KD-9 did not show any GIC shift with regard to RIF when cultured with or without ATc inducer.

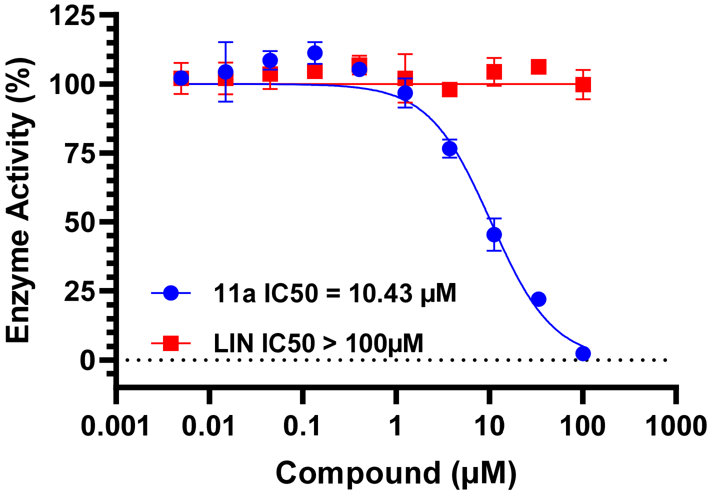


**Figure S7.** Biochemical analysis of LIN against recombinant human glutamine synthetase (GS). Human GS purchased from Novus Biologicals (www.novusbio.com, cat no. NBP2-52619), was active as measured by the malachite green assay (specific activity >2,800 pmol/min/ug). LIN does not inhibit the human GS at 100 µM in the assay while 11a inhibits the human GS at $IC_{50}$ ~10.43 µM.
